# Bcl-2, via Its BH4 Domain, Blocks Apoptotic Signaling Mediated by Mitochondrial Ras

**DOI:** 10.1074/jbc.M210202200

**Published:** 2002-12-10

**Authors:** Gerald V. Denis, Qiang Yu, Peihong Ma, Linda Deeds, Douglas V. Faller, Chang-Yan Chen

**Affiliations:** Cancer Research Center and Department of Medicine, Boston University School of Medicine, Boston, Massachusetts 02118

## Abstract

Bcl-2 protects cells against Ras-mediated apoptosis; this protection coincides with its binding to Ras. However, the protection mechanism has remained enigmatic. Here, we demonstrate that, upon apoptotic stimulation, newly synthesized Bcl-2 redistributes to mitochondria, interacts there with activated Ras, and blocks Ras-mediated apoptotic signaling. We also show, by employing *bcl-2* mutants, that the BH4 domain of Bcl-2 binds to Ras and regulates its anti-apoptotic activity. Experiments with a C-terminal-truncated Ras or a farnesyltransferase inhibitor demonstrate that the C*AAX* motif of Ras is essential for apoptotic signaling and Bcl-2 association. The results indicate a potential mechanism by which Bcl-2 protects cells against Ras-mediated apoptotic signaling.

Bcl-2 suppress apoptosis elicited by various pro-death stimuli (1–3). Bcl-2 family members include anti- and pro-apoptotic factors that possess hydrophobic stretches of amino acids at the C termini that allow them to bind to intracellular membranes, including the membranes of ER^1^ and of mitochondria (4–6). Bcl-2 and most of its homologs share four conserved regions: Bcl-2 homology domains (BH1–4), through which the proteins dimerize with each other or with other proteins (7–11). The BH1 and BH2 domains of Bcl-2 are required for homodimerization with the pro-apoptotic Bcl-2 family member Bax. The BH3 domain of some members of the Bcl-2 family (BH3-only proteins, such as Bad, Bik, or Bid) interacts with other Bcl-2 proteins to initiate apoptosis (12, 13). The BH4 domain, a heterodimerization region, is responsible for Bcl-2-directed targeting of Raf-1 to mitochondria and the cooperation between Bcl-2 and Raf-1 in the suppression of apoptosis (50). The loop region of Bcl-2 regulates its protective function (14). Bcl-2 that lacks its C-terminal domain inefficiently associates with cellular membranes and is incapable of preventing apoptosis (15, 16). We previously demonstrated that overexpressed Bcl-2 prevents cells that express v-Ha-*ras* from undergoing apoptosis in response to protein kinase C (PKC) down-regulation. This protection coincides with the binding of Bcl-2 to Ras (17, 18). However, the molecular mechanism by which the interaction of Bcl-2 and Ras regulates apoptosis has remained unknown.

The biological effects of Ras on cell growth or apoptosis depend strongly upon the kind of stimulus, cell type, or regulatory environment (19). Human or mouse lymphocytes expressing activated *ras* undergo apoptosis in response to PKC down-regulation and this apoptotic process is blocked by overexpression of *bcl-2* (17, 18, 20). Ras activity is involved in Fas-regulated, multiple signaling pathways (13, 21). However, Bcl-2 partially protects lymphocytes from Fas-induced apoptosis, possibly through decreasing the permeability of the mitochondrial membrane (22, 23). It is well known that upon activation or mitogenic stimulation a series of post-translational modifications, including prenylation, -*AAX* proteolysis, and carboxyl methylation, occur on the C terminus of Ras. This process allows Ras to associate with membrane compartments, especially with plasma membrane (24–26). Ras family proteins (Ki-, Ha-, and N-Ras) have also been demonstrated to associate with mitochondria in murine lymphokine-dependent TS1*αβ* cells (27). The function of mitochondrial Ras has not been fully investigated yet. Furthermore, the C*AAX* motif of Ras may regulate its N-terminal conformation and further affect its ability to interact with other proteins (2, 26, 28). It is, so far, unclear whether prenylation on the C*AAX* motif of Ras is required for its apoptotic signaling.

In Fas-induced apoptosis, endogenous Ras in Jurkat cells is activated via the ceramide signaling pathway (22, 23, 29). Jurkat cells have been reported to be sensitive to Fas-engagement, which may be due to very low expression of Bcl-2 (18, 30). In order to explore further the mechanism(s) of Bcl-2 protection against Ras-mediated apoptotic signaling, we introduced either *bcl-2* or *ras*, as well as both genes, into Jurkat cells, and then examined the consequences in the setting of Fas-engagement or PKC suppression. We demonstrate that, under apoptotic conditions, newly synthesized Bcl-2 is preferentially expressed in mitochondria, and subsequently binds to activated, mitochondrial Ras. Such regulation was confirmed in EBV-positive Burkitt’s lymphoma Akata cells that express increased levels of endogenous Bcl-2 or mouse lung cancer LKR cells that contain activated *ras*. Also, by using various *bcl-2* mutants, we identified the BH4 domain of Bcl-2 as the interaction site with Ras, and this domain is crucial for protection against Ras-mediated apoptotic signaling. Furthermore, we established that the integrity of the C*AAX* motif of Ras and its prenylation are necessary for apoptotic activity, and regulate the ability of Ras to associate with Bcl-2.

## EXPERIMENTAL PROCEDURES

### Cell Lines and Transfection—

Jurkat cells were obtained from ATCC. Fas/FADD-defective Jurkat cells were generated by random mutagenesis and obtained from Dr. J. Blenis (Harvard Medical School, MA). The EBV-positive Burkitt’s lymphoma Akata cell line and the EBV-negative Burkitt’s lymphoma Ramos cell line were obtained from Dr. S. Ghosh (Boston University School of Medicine). The lung cancer LKR cells derived from the lung foci of v-Ki-*ras* transgenic mouse were given by Dr. T. Jack (MIT). v-Ha-*ras* or *ras* with a C-terminal deletion was inserted into a *MSCV* retroviral vector containing the *neo* gene (Invitrogen). The expression of *ras* was examined by Northern blot (17). *bcl-2* and various *bcl-2* mutants were generously provided by Dr. T. Parslow (University of California at San Francisco). An ER marker and a c-*myc* epitope (pCMV/*myc*/ER) were inserted into a pShooter vector for the determination of ER location (Invitrogen).

### Preparation of Various Cellular Fractions—

Cells (50 × 10^6^), after the treatments, were washed twice with 1× phosphate-buffered saline and resuspended in 1 ml of 1% Triton X-114 lysis buffer (31). The cell suspensions were transferred to a 1-ml syringe and sheared by being passed 40 times through a 25-gauge needle. The lysates were centrifuged at 280 × *g* for 10 min to precipitate nuclei, and the supernatants were collected. One-third of the whole cell extract was saved, and the remainder was centrifuged at 16,000 × *g* for 30 min. The supernatant (cytosol) was collected, and the pellet was washed in the lysis buffer containing 1% Nonidet P-40 for 1 h on ice and centrifuged again at 100,000 × *g*. The supernatant (P100) was saved (50). For the ER fraction, an OptiPrepTM kit (Invitrogen) was used. For the mitochondrial fraction, 3 × 10^9^ cells were resuspended in buffer A (50 mm Tris, pH 7.5, 1 mm EGTA, 5 mm 2-mercaptoethanol, 0.2% bovine serum albumin, 10 mm KH_2_PO_4_, pH 7.6, 0.4 m sucrose), and allowed to swell on ice for 40 min. After differential centrifugation, the resulting pellets were resuspended in buffer B (10 mm KH_2_PO_4_, pH 7.2, 0.3 mm mannitol, 0.1% bovine serum albumin). Mitochondria were subsequently separated on a sucrose step gradient (32).

### Immunoprecipitation and Immunoblot—

After treatment with anti-Fas Ab (1.5 *μ*g/ml for 60 min for human cells (PanVera Corp.) or HMG (1-*O*-hexadecyl-2-*O*-methyl-*rac*-glycerol, 150 nm for 24 h, Calbiochem), cell fractions were isolated and total protein concentrations in each fraction were normalized. Subsequently, the fractions were adjusted to 0.4 m NaCl, 0.5% deoxycholate, and 0.05% SDS (31). Each sample was divided into two aliquots for reciprocal immunoblotting. The samples were immunoprecipitated with either anti-pan-Ras Ab (Oncogene Science) or anti-human Bcl-2 Ab (BD PharMingen) for 4 h at 4 °C. Immunoprecipitates were collected with protein A-Sepharose and separated on a 12% SDS-PAGE gel. Bcl-2 or Ras were then detected with anti-Ras Ab or anti-Bcl-2 Ab. Anti-cytochrome *c*, inositol trisphosphate receptor (IP_3_R) or tubulin Abs (Santa Cruz Biotechnology Inc.) were used as immunoblot controls.

### Flow Cytometry Analysis—

For cell surface staining, the cells were incubated with anti-Fas Ab for 2 h and stained with a second Ab conjugated with fluorescein. For DNA fragmentation assay, after the treatments with anti-Fas Ab [1.5 *μ*g/ml for 60 min for human cells (PanVera Corp.) or HMG (1-*O*-hexadecyl-2-*O*-methyl-*rac*-glycerol, 150 nm for 24 h, Calbiochem), cells (1 × 10^6^/ml) were washed with phosphate-buffered saline twice, fixed with 70% ethanol, and stained with phosphate-buffered saline containing 10 ng/ml RNase and 50 ng/ml propidium iodide.

## RESULTS

**Fig. 1.**
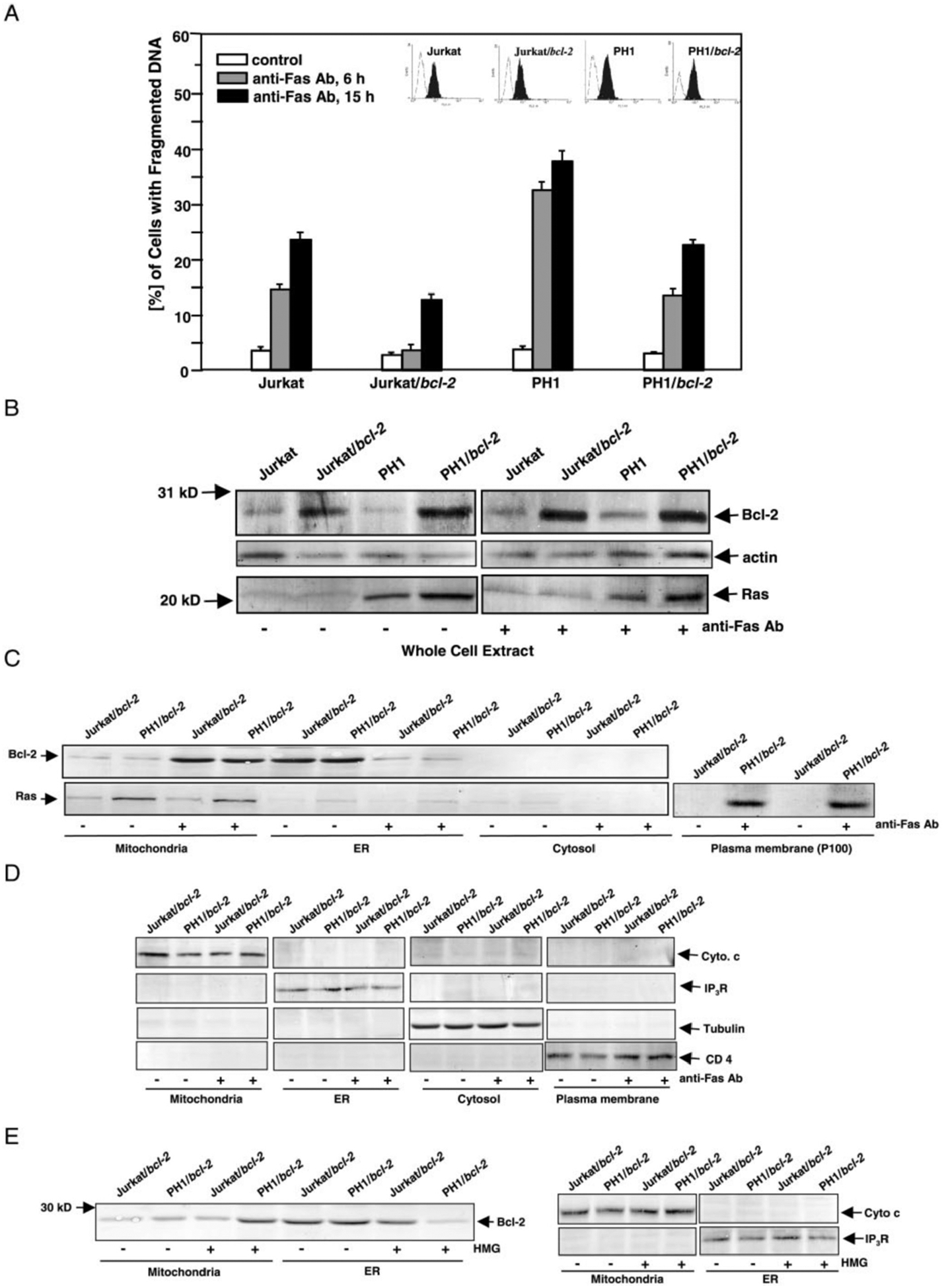
The expression of Bcl-2 or Ras upon apoptotic stimulation. *A*, effect of overexpressed Bcl-2 on Fas-mediated apoptosis. The cells were untreated or treated with anti-Fas Ab for 6 or 15 h, and stained with propidium iodide. The percentages of the cells with fragmented DNA were determined by flow cytometry. The *error bars* represent S.D. of five independent experiments. *Inset*, cells were stained with anti-Fas Ab and subsequently with anti-mouse IgG Ab conjugated to fluorescein for determination of surface Fas Ag expression. *Unshaded profile*, cells stained with the second Ab alone; *dark profile*, cells stained with anti-Fas Ab plus the second Ab. *B*, Bcl-2 or Ras expression in whole cell extract. The cells were untreated or treated with anti-Fas Ab for 60 min, and whole cell extracts were then prepared. Samples containing equal amounts of total proteins were separated on a 12% SDS-PAGE gel and then probed with anti-Bcl-2 or Ras Ab. *C*, Bcl-2 or Ras expression in various subcellular compartments. The mitochondrial, ER, cytosolic, or plasma membrane fractions from Jurkat/*bcl-2* or PH1/*bcl-2* cells, with or without Fas-ligation, were prepared. Expression of Bcl-2 or Ras in these subcellular fractions was determined by immunoblotting. *D*, relative purity of subcellular fractions, with or without Fas-ligation, was determined with anti-cytochrome *c*, -IP3R, -tubulin, or -CD4 Ab. *E*, subcellular localization of Bcl-2 in PKC/Ras-mediated apoptosis. After exposure of cells to HMG (150 nM) for 24 h, the mitochondrial or ER fractions were prepared. Expression of Bcl-2 was examined by immunoblotting with anti-Bcl-2 Ab (*left panel*). Relative purity of subcellular fractions was determined with the corresponding antibodies (*right panels*).

We previously demonstrated that oncogenic *ras* elicits apoptosis once the activity of endogenous PKC is suppressed, and that the overexpression of Bcl-2 blocks this process, possibly through its association with Ras (18). The mechanism of Bcl-2-mediated protection against Ras apoptotic signaling is unclear. Because Ras is involved in Fas-induced apoptosis and Bcl-2 partially protects cells from Fas-induced apoptosis (20, 22, 23, 39, 33), we tested whether Bcl-2 interacts with Ras during Fas-induced apoptosis. v-Ha-*ras*, *bcl-2*, or both genes were introduced into Jurkat cells (designated PH1, Jurkat/*bcl-2*, or PH1/*bcl-2*, respectively), and a DNA fragmentation assay was conducted. The cells were treated with anti-Fas antibody (Ab) for 6 or 15 h to engage Fas signaling, and the percentages of the cells with fragmented DNA were analyzed by flow cytometry (Fig. 1A). Jurkat cells that overexpress v-Ha-*ras* (PH1 cells) were more susceptible to Fas-induced apoptosis than the other three cell lines. The percentage of PH1 cells with fragmented DNA was dramatically increased at 6 h after addition of anti-Fas Ab (about 35%), and reached more than 40% after 15 h, indicating that overexpressed, activated Ras accelerates Fas-induced apoptosis. However, DNA fragmentation in Jurkat/*bcl-2* cells did not increase 6 h after Fas-ligation and was only about 12% at 15 h. The level of DNA fragmentation in PH1/*bcl-2* cells was intermediate between the levels observed in Jurkat/*bcl-2* and PH1 cells. It appears that overexpressed Bcl-2, in the initial period (up to 15 h after Fas-ligation), protects cells (Jurkat/*bcl-2*) from Fas-mediated cell death, or delays the onset of the apoptotic process. A control experiment was also conducted using an unrelated, isotype-matched Ab. An unrelated Ab did not cause apoptosis (data not shown). Indirect immunofluorescence staining of Fas was also conducted to exclude the possibility that introduction of the exogenous gene(s) affects surface expression of Fas antigen. The levels of Fas did not change if *bcl-2*, *ras* or both were overexpressed (Fig. 1A, *inset*).

We then examined whether Fas-engagement alters Bcl-2 or Ras protein expression using immunoblotting analysis. Jurkat or PH1 cells expressed very low amounts of Bcl-2. The levels of Bcl-2 in a whole cell extract from untreated Jurkat/*bcl-2* and PH1/*bcl-2* cells were higher (about 6.6-fold than Jurkat or PH1 cells), and Fas-ligation did not cause further induction of the protein (Fig. 1B, *upper panels*). A Ras immunoblot showed a similar result, in which the *ras* transfectants express 5-fold higher levels of the protein compared with the parental cell lines (Fig. 1B, *lower panels*). Again, Fas-ligation did not alter protein expression. Because the parental cells express a very low amount of Bcl-2 or Ras, we used Jurkat/*bcl-2* and PH1/*bcl-2* cells to examine whether Fas-ligation alters the subcellular distribution of Bcl-2 and Ras. The subcellular fractions from these two cell lines, with or without Fas-ligation, were prepared for immunoblotting (Fig. 1C). Bcl-2 was detectable in the mitochondrial fraction of untreated cells, and the protein level increased 4–6-fold in response to Fas-engagement. Significant Bcl-2 was seen in the ER fraction under normal growth conditions. After Fas-ligation, however, the level of Bcl-2 in ER was dramatically reduced (about 3–4-fold). Bcl-2 was not detected in the cytosol fractions from these cells, as expected. The expression of Ras in the subcellular fractions was also examined by immunoblot. A basal level of endogenous Ras protein was detected in various subcellular membrane fractions from Jurkat/*bcl-2* cells under normal growth conditions, and Fas-ligation did not alter the protein expression. In contrast, moderate and high levels of Ras protein were detected in the mitochondrial and plasma membrane fractions from untreated PH1/*bcl-2* cells, respectively, and the expression did not change upon Fas-engagement. The data suggest that apoptotic stimulation by Fas-ligation alters the expression of Bcl-2 in the subcellular compartments, but not of Ras. The relative purity of the subcellular fractions from the cells, with or without Fas-engagement, was confirmed by immunoblotting for the expression of cytochrome *c* (a mitochondrial marker), inositol trisphosphate receptor (IP_3_R, an endoplasmic reticulum marker), tubulin (a cytosol marker), and CD4 (a plasma membrane marker) respectively (Fig. 1D). After the apoptotic stimulation, equal amounts of the proteins were detected in their corresponding subcellular fractions in comparison to the untreated controls. Bcl-2 expression in various subcellular fractions was also examined in different *bcl-2* or *bcl-2/ras* clones, and similar results were obtained, which excludes the possibility of clonal variation (data not shown).

We next examined whether changes of Bcl-2 expression levels in different subcellular fractions occur in PKC/Ras-mediated apoptosis. Jurkat/*bcl-2* and PH1/*bcl-2* cells were exposed to HMG (150 nM, a PKC inhibitor) for 24 h, and then the mitochondrial and ER fractions were prepared for immunoblotting (Fig. 1E, *left panel*). In response to PKC inhibition, the level of Bcl-2 in the ER fraction from PH1/*bcl-2* cells was dramatically reduced, which coincided with increased protein in mitochondria. Interestingly, because PKC suppression is not a death signal to Jurkat/*bcl-2* cells, the levels of the protein in the subcellular fractions from the cells did not change, which suggests that the altered expression of Bcl-2 in mitochondria of PH1/*bcl-2* cells after PKC suppression is related to apoptosis. The relative purity of the subcellular fractions from the cells, with or without HMG treatment, was confirmed by the immunoblotting (Fig. 1E, *right panels*).

**Fig. 2.**
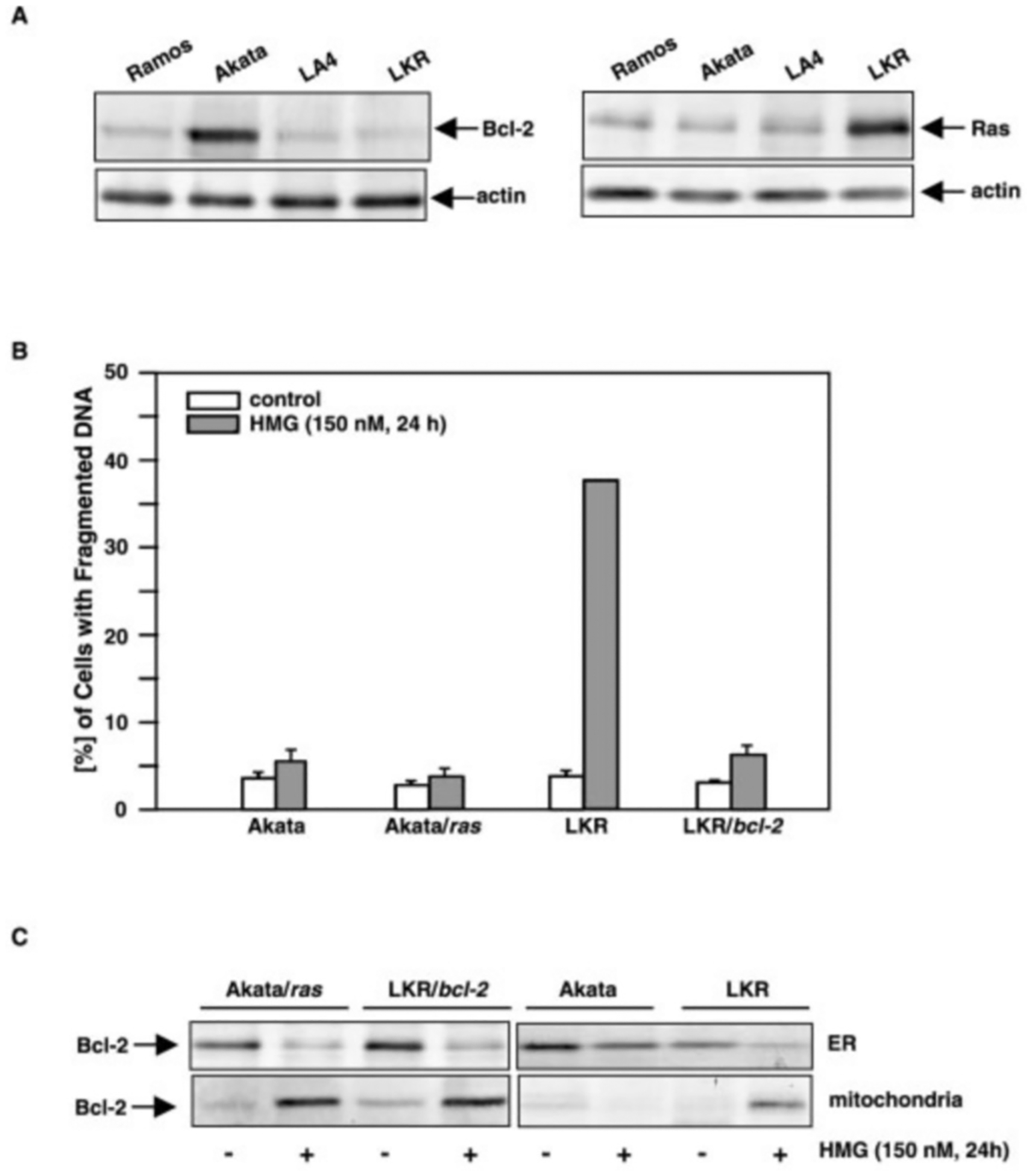
The effect of endogenous Bcl-2 or Ras on PKC/Ras-mediated apoptosis. *A*, cell lysates from human Burkitt’s lymphoma Ramos and Akata cells, or mouse lung epithelial-like LA4 cells and mouse lung cancer LKR cells were prepared. The expression of Bcl-2 or Ras in the lysates was determined by immunoblotting (*upper panels*). The equal loading of total proteins in the lysates was defined by re-probing the blots with anti-actin Ab. *B*, after introducing *ras* into Akata cells or *bcl-2* into LKR cells, the cells were treated with HMG (150 nM) for 24 h, and then stained with propidium iodide. The percentages of the cells with fragmented DNA were determined by flow cytometry. The *error bars* represent the S.D. of five independent experiments. *C*, subcellular localization of endogenous Bcl-2 in PKC/Ras-mediated apoptosis. After treating the cells with HMG (150 nM) for 24 h, the mitochondrial or ER fractions were prepared. Expression of Bcl-2 was examined by immunoblotting with anti-Bcl-2 Ab (*left panels*). Relative purity of subcellular fractions was determined with the corresponding antibodies (*right panels*).

It is known that Bcl-2 is induced in some Epstein-Barr virus (EBV)-positive Burkitt’s lymphoma cells (34). Akata (a EBV-positive) and Ramos (a EBV-negative) Burkitt’s cell lines were used to test whether endogenous Bcl-2 could provide the same protection. We also employed mouse LA4 cells (a mouse lung epithelial-like cell line) and LKR (a mouse lung cancer cell line derived from the lung foci of v-Ki-*ras* transgenic mouse). The expression of Bcl-2 or Ras in these cells was examined by immunoblotting (Fig. 2A, *upper panels*). Increased amounts of Bcl-2 or Ras were detected in Akata or LKR cells, respectively. The amount of total proteins in each sample was monitored by re-probing the blots with anti-actin Ab (Fig. 2A, *lower panels*). We then introduced v-Ha-*ras* or *bcl-2* into Akata (Akata/*ras*) or LKR (LKR/*bcl-2*) cells to examine their susceptibility to PKC/Ras-mediated apoptosis, using a DNA fragmentation assay. After treating the cells with HMG, the percentages of the cells with fragmented DNA were analyzed by flow cytometry (Fig. 2B). The inhibitor did not induce apoptosis in Akata cells, which is consistent with our previous observation in which normal cells (without oncogenic *ras*) do not die in response to PKC suppression (17, 18). However, the introduction of v-*ras* did not render the cells (Akata/*ras*) susceptible to HMG-mediated cell killing, indicating that overexpression of endogenous Bcl-2 blocks the apoptotic process. In comparison, about 40% of mouse lung cancer LKR cells had fragmented DNA after HMG treatment. Overexpressed *bcl-2* interfered with this apoptotic process in LKR/*bcl-2* cells. The expression of Bcl-2 in different subcellular compartments of the cells was also examined (Fig. 2C, *left panels*). The ER and mitochondrial fractions from the cells with or without HMG treatment were prepared and subsequently immunoblotted with an anti-Bcl-2 Ab. A high level of Bcl-2 was detected in the ER fraction from untreated Akata, Akata/*ras*, and LKR/*bcl-2* cells, and the protein was moderately expressed in the ER fraction from LKR cells. Bcl-2 in the ER was significantly reduced after HMG treatment in Akata/*ras*, LKR/*bcl-2*, and LKR cells, but not in Akata cells. In mitochondria, a very low amount of the protein was detected in the untreated cells. However, the protein level in those cells (except Akata cells) was dramatically increased in response to HMG treatment. The data, again, indicate that endogenous Bcl-2 redistributes and protects the cells against PKC/Ras-mediated apoptosis. The relative purity of the subcellular fractions from the cells, with or without Fas-engagement, was confirmed by immunoblotting (Fig. 2C, *right panels*).

**Fig. 3.**
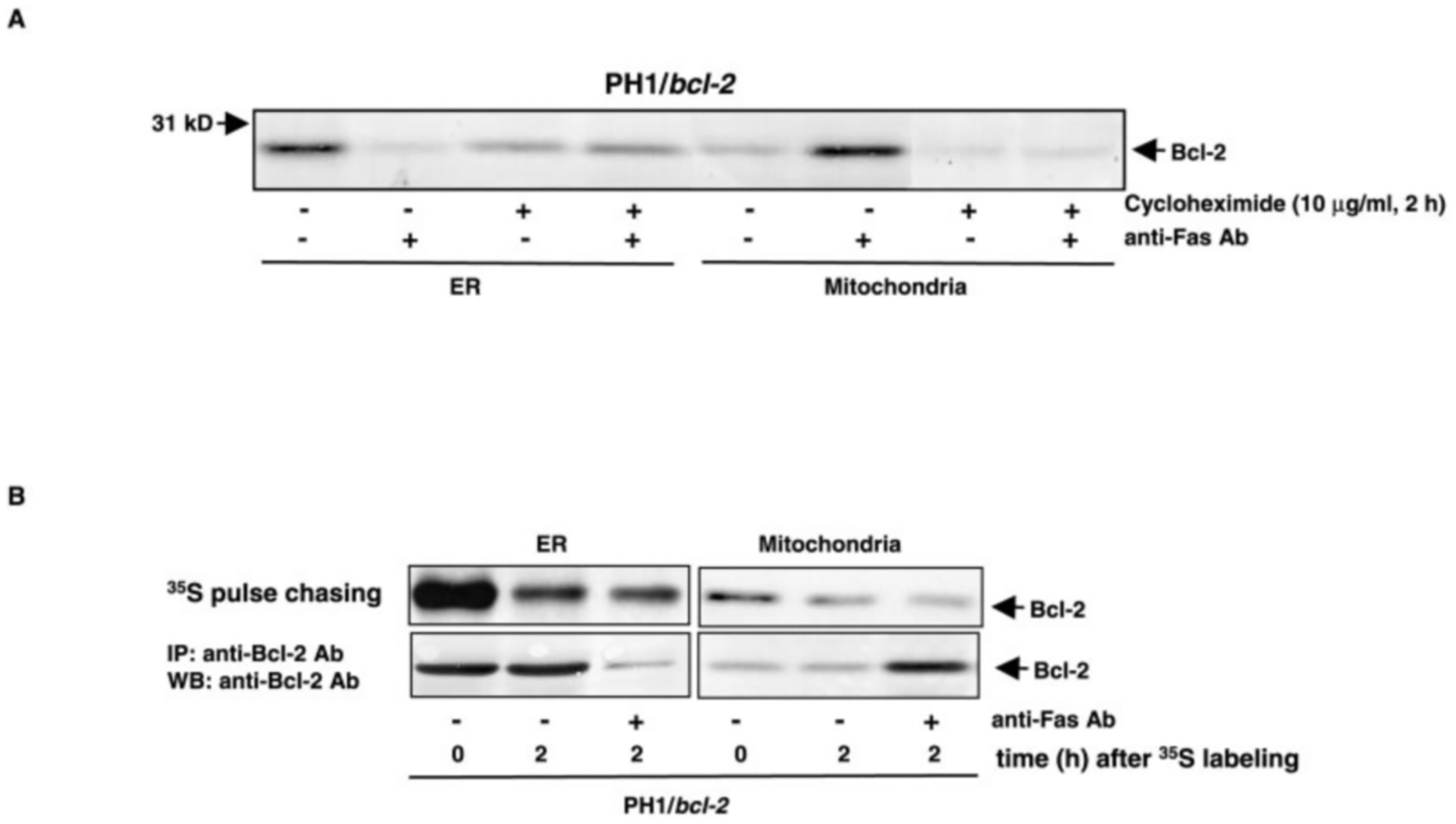
Association of Bcl-2 with mitochondria after Fas ligation. *A*, effect of protein synthesis inhibitor on Bcl-2 expression. PH1/*bcl-2* cells were exposed to cycloheximide (a protein synthesis inhibitor), prior to Fas-engagement. Subsequently, the ER and mitochondrial fractions from the cells were prepared for immunoblotting using an anti-Bcl-2 Ab. *B*, [^35^S]methionine labeling and co-immunoprecipitation and immunoblotting of Bcl-2. After culturing in methionine-free medium for 6 h, PH1/*bcl-2* cells were grown in the medium containing [^35^S]methionine for 2 h. With or without Fas-ligation, the ER or mitochondrial fraction were prepared at 0 and 2 h after the termination of the labeling, and each sample was divided into two equal portions for ^35^S pulse-chasing (*upper panels*) and co-immunoprecipitation and immunoblotting (*lower panels*).

We then tried to determine whether the altered expression of Bcl-2 in the subcellular fractions during apoptosis is due to the translocation or redistribution of the protein. After Fas-ligation, immunoblotting of ER or mitochondrial Bcl-2 in PH1/*bcl-2* cells, with or without cycloheximide (a protein synthesis inhibitor) treatment, was performed (Fig. 3A). Bcl-2 was visualized in the ER fraction under normal growth conditions, and only a small amount of the protein was detected in mitochondria. After Fas-ligation, the level of Bcl-2 in ER was dramatically reduced, and, in contrast, the protein expression in mitochondria was augmented in the cells. After blocking protein synthesis by cycloheximide, the amount of ER Bcl-2, with or without Fas-engagement, was reduced to more than half in comparison to the control. In the mitochondrial fraction, in the presence of cycloheximide, the protein was decreased to an almost undetectable level. The data suggest that the increased amount of mitochondrial Bcl-2 induced by Fas-ligation (seen in Fig. 1C) is from the newly synthesized protein, but not from pre-existing Bcl-2 via translocation. To confirm this further, ^35^S pulse-chasing analysis was conducted (Fig. 3B). After labeling PH1/*bcl-2* cells with [^35^S]methionine, the ER and mitochondrial fractions were prepared. Each sample was divided into two portions for ^35^S pulse-chasing or co-immunoprecipitation and immunoblotting. Two hours after terminating the pulse, ^35^S-labeled Bcl-2 in ER, with or without Fas Ab treatment, was reduced (about 0.7-fold reduction) (Fig. 3B, *upper panels*). In the mitochondrial fraction, a similar decay pattern of labeled Bcl-2 was also observed. In a co-immunoprecipitation and immunoblotting experiment, Bcl-2 expression in the ER fraction was dramatically reduced after Fas-ligation, and the mitochondrial Bcl-2 expression was increased (Fig. 3B, *lower panels*). Therefore, the results provide the same conclusion that newly synthesized Bcl-2, upon Fas-engagement, is preferentially expressed in mitochondria.

**Fig. 4.**
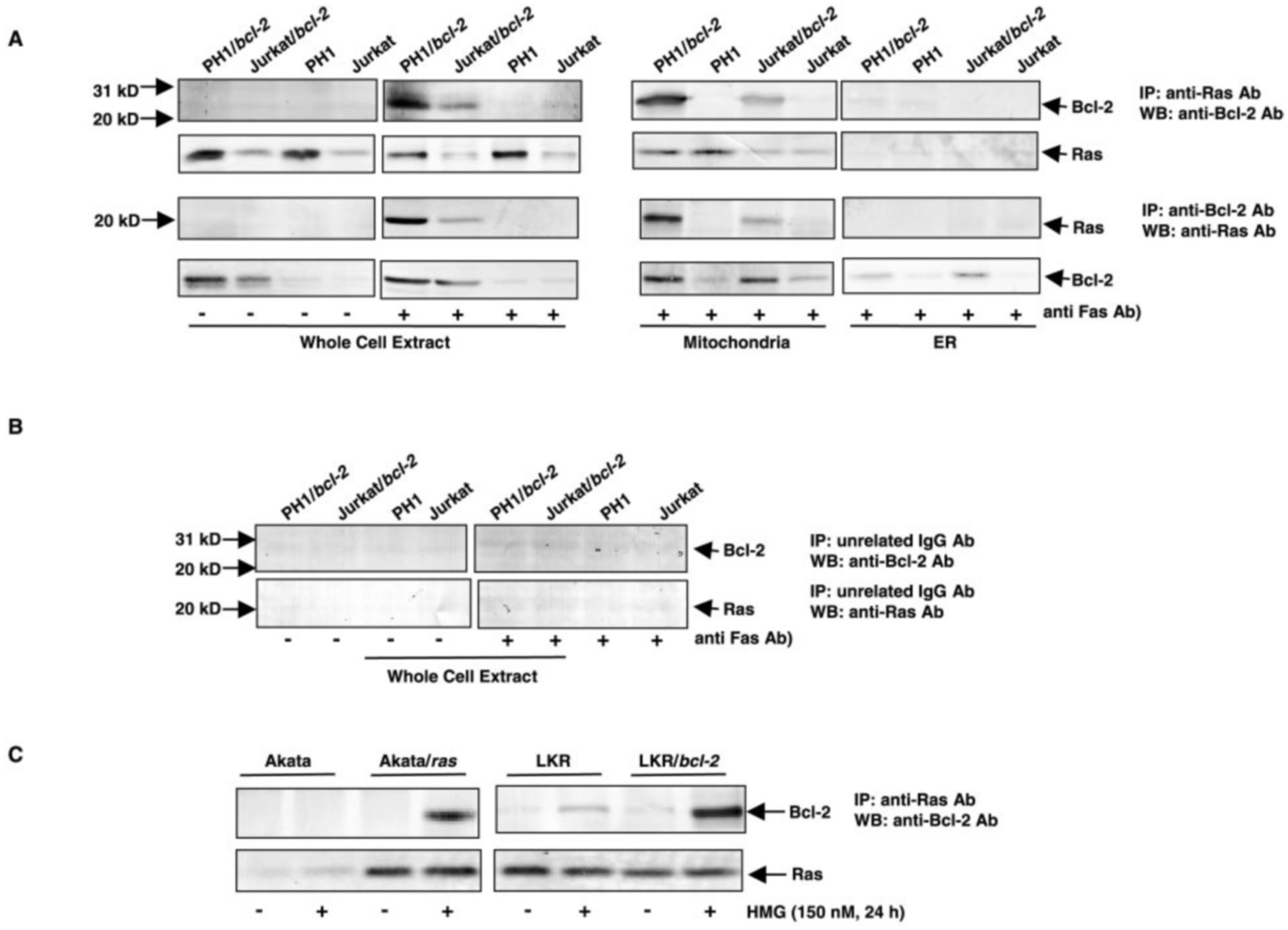
Interaction of Bcl-2 and Ras. *A*, whole cell extracts and mitochondrial or ER fractions from the cells, with or without Fas-ligation, were prepared and immunoprecipitated with anti-Ras Ab or anti-Bcl-2 Ab. Subsequently, the immunoprecipitates were examined for the presence of Bcl-2 (*first row*) or Ras (*third row*) by immunoblotting with the corresponding Abs. The same blots were also re-probed with the same Ab as that used in the immunoprecipitation (*second* and *fourth rows*). *B*, whole cell extracts from the cells, with or without Fas-ligation, were immunoprecipitated with preimmune serum, and then immunoblotted with anti-Bcl-2 or Ras Ab. *C*, whole cell lysates from Akata, Akata/*ras*, LKR, and LKR/*bcl-2* cells were prepared. Subsequently, the immunoprecipitation using anti-Ras Ab and immunoblotting with anti-Bcl-2 Ab was performed (*upper panels*). The same blots were re-probed with the same Ab as that used in the immunoprecipitation (*lower panels*).

We previously demonstrated that, in PH1/*bcl-2* cells, Bcl-2 bound to Ras in response to PKC down-regulation (18). Here, we tried to confirm further this association by using another apoptotic setting (Fas-engagement) and to determine the location of the interaction. The reciprocal co-immunoprecipitation of Bcl-2 and Ras was performed (Fig. 4). There was no co-precipitation of these two molecules in the whole cell lysate from untreated Jurkat, Jurkat/*bcl-2*, PH1, or PH1/*bcl-2* cells (Fig. 4A, *first row*). After Fas-ligation, an anti-Bcl-2 immunoblot visualized Bcl-2 associated with the anti-Ras co-immunoprecipitate from PH1/*bcl-2* and Jurkat/*bcl-2* cells. The association was not seen in either PH1 or Jurkat cells, which is probably due to the fact that Jurkat cells express very low amounts of Bcl-2 and Ras. The similar result was obtained from the co-immunoprecipitation and immunoblotting of the mitochondrial fraction, in which a high level of Bcl-2 was recovered in the anti-Ras co-immunoprecipitate from PH1/*bcl-2* cells after Fas-ligation, and a lower level of the protein was detected in Jurkat/*bcl-2* cells. The interaction was not detected in the ER fractions from any of the four cell lines after Fas-engagement. In a reciprocal experiment, wherein the initial immunoprecipitation was carried out with anti-Bcl-2 Ab, an anti-Ras immunoblot detected an increased amount of Ras in the co-immunoprecipitate from the whole cell extract or mitochondrial fraction of PH1/*bcl-2* cells, and a detectable level of co-bound Ras in the same subcellular fractions of Jurkat/*bcl-2* cells, in response to Fas-ligation (Fig. 4A, *third row*). Again, Ras did not co-immunoprecipitate with the anti-Bcl-2 Ab in ER fractions from any of the four cell lines in response to Fas-engagement. The presence of Ras (Fig. 4A, *second row*) or Bcl-2 (Fig. 4A, *fourth row*) in the immunoprecipitates from these subcellular fractions was also confirmed by re-probing the corresponding blots with either the anti-Ras Ab or the anti-Bcl-2 Ab. As a control, preimmune serum was used for immunoprecipitation in whole cell extracts from the cells, followed by immunoblotting with anti-Bcl-2 or anti-Ras Ab to eliminate the possibility of nonspecific binding of these two proteins (Fig. 4B). There was no co-immunoprecipitation of either Bcl-2 or Ras with preimmune serum. The same results were obtained from the mitochondrial fraction (data not shown). The co-immunoprecipitation and immunoblotting of Bcl-2 and Ras, in response to PKC suppression, was also performed in Akata, Akata/*ras*, LKR, and LKR/*bcl-2* cells (Fig. 4C, *upper panels*). The strong interaction between Bcl-2 and Ras, after HMG treatment, was detected in Akata/*ras* or LKR/*bcl-2* cells. A low level of Bcl-2 was precipitated by the anti-Ras Ab in LKR cells. The association was not detected in Akata cells in response to the treatment (because PKC inhibition is not a death signal to the cells). The existence of Ras in the co-precipitates was confirmed by re-probing the same blot with the anti-Ras Ab (Fig. 4C, *lower panels*). Overall, the data suggest that the physical association of Bcl-2 with Ras occurs only under apoptotic conditions and in mitochondria.

**Fig. 5.**
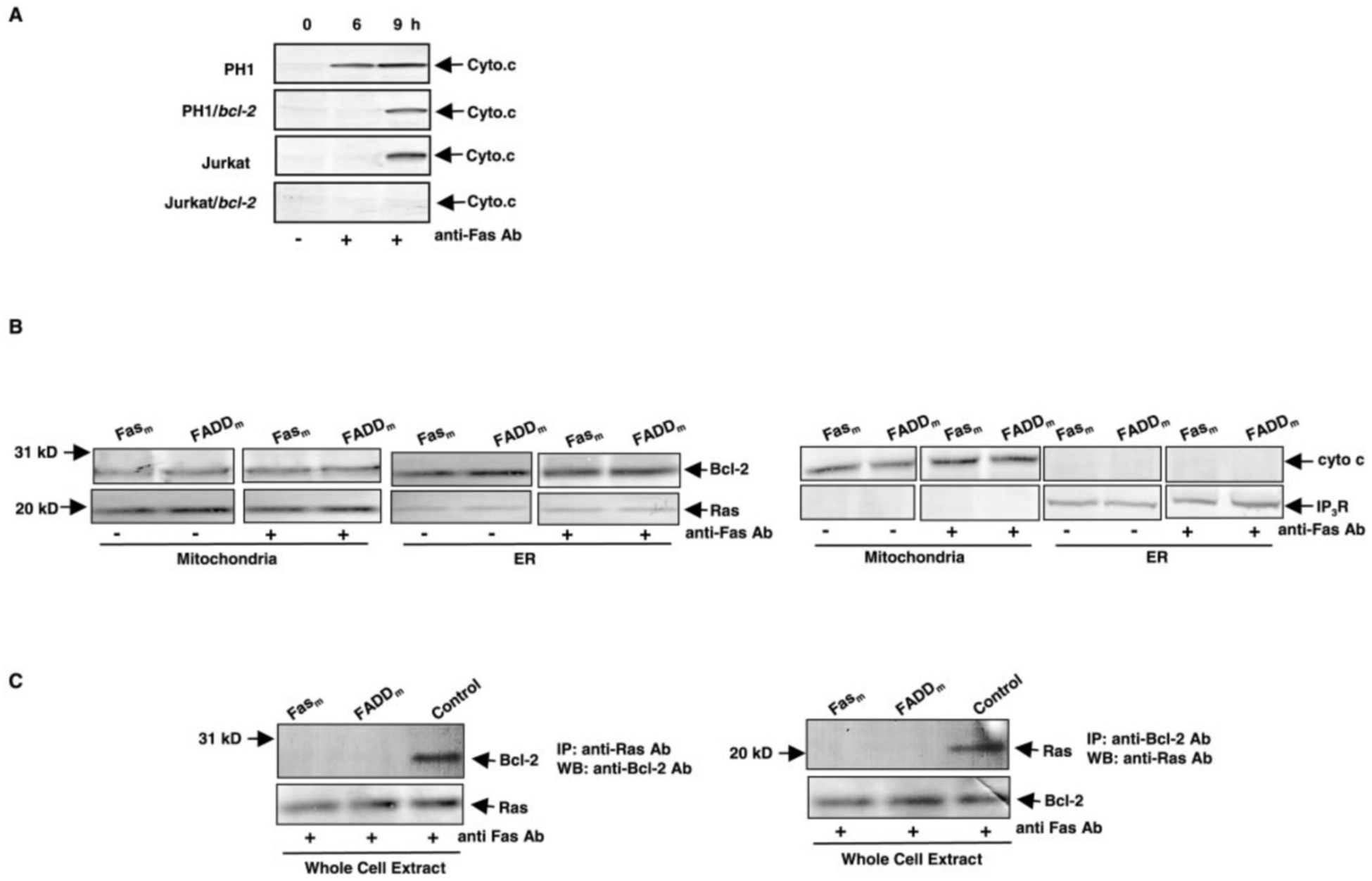
Bcl-2 redistribution and its Ras interaction requires Fas signaling. *A*, cytochrome *c* release mediated by Fas-ligation in Jurkat cells that express *bcl-2*, *ras* or both. PH1, PH1/*bcl-2*, Jurkat, or Jurkat/*bcl-2* cells were untreated or treated with anti-Fas Ab for 6 or 9 h, and the cytosolic fractions were prepared. The samples, containing equal amounts of total proteins were separated on a 15% SDS-PAGE gel, transferred to a nitrocellulose, and immunoblotted with anti-cytochrome *c* Ab. *B*, Bcl-2 or Ras distribution in Fas or FADD mutant cells. The mitochondrial and ER fractions from Jurkat/Fas_m_ and Jurkart/FADD_m_ cells that co-express v-Ha-*ras* plus *bcl-2*, with or without Fas-ligation, were prepared. The expression of Bcl-2 or Ras in these subcellular fractions was determined by immunoblotting (*left panels*). Relative purity of subcellular fractions was determined with the corresponding antibodies (*right panels*). *C*, interaction of Bcl-2 and Ras in Fas- or FADD-deficient cells. The whole cell extracts prepared from the mutant cells co-expressing *ras* and *bcl-2*, after 60 min of Fas-ligation, were immunoprecipitated with anti-Ras Ab or anti-Bcl-2 Ab. The immunoprecipitates were examined for the presence of Bcl-2 or Ras by immunoblotting with the corresponding Abs (*upper panels*). The control represents Jurkat cells co-expressing *ras* and *bcl-2* (PH1/*bcl-2*). The same blots were also re-probed with the same Ab as that used in the immunoprecipitation (*lower panels*).

Cytochrome *c* release indicates an increase in mitochondrial permeability, and Bcl-2 regulates the permeability transition to block apoptosis (35–37). We tested if overexpressing *ras*, *bcl-2*, or both genes affects mitochondrial cytochrome *c* release to cytosol after Fas-engagement (Fig. 5A). Cytoplasmic fractions from PH1, PH1/*bcl-2*, Jurkat and Jurkat/*bcl-2* cells at various times after Fas-engagement were prepared for immunoblot analysis. Cytochrome *c* was detected in the cytosol fraction from PH1 cells 6 h after Fas-ligation. Release of the protein was persistent during the apoptotic process. In Jurkat/*bcl-2* cells, the protein was undetectable in the cytosol 9 h after addition of anti-Fas Ab, and cytosolic cytochrome *c* did appear in PH1/*bcl-2* cells. The pattern of cytochrome *c* release in Jurkat cells was similar to PH1/*bcl-2* cells. The data suggest that the anti-apoptotic effects of *bcl-2* and the pro-apoptotic effect of *ras* both contribute to the degree of mitochondrial damage resulting from Fas-induced apoptotic signaling. The relative purity of the cytosolic fractions from the cells, with or without Fas-engagement, was confirmed by immunoblotting (data not shown).

The formation of the death-inducing signaling complex (DISC) via recruitment of FADD and caspase 8 is the initial event in Fas signaling (38). To confirm that the redistribution of Bcl-2 and its interaction with Ras indeed depend upon an intact DISC, v-Ha-*ras* and *bcl-2* were co-introduced into Fas or FADD mutant Jurkat cells (Fas_m_ or FADD_m_ cells). The defect in the formation of DISC (which is due to Fas or FADD mutation) impaired the redistribution of Bcl-2 mediated by Fas-ligation (Fig. 5B, *left panels*). Ras expression in the mutant cells did not change and was the same as in the non-mutant cells (PH1/*bcl-2*), as expected. The relative purity of the subcellular fractions from the cells, with or without Fas-engagement, was confirmed by immunoblotting for cytochrome *c* or IP3R, respectively (Fig. 5B, *right panels*). Reciprocal co-immunoprecipitation and immunoblotting experiments, after Fas-ligation, were also performed with whole cell extracts from the mutant cells as well as PH1/*bcl-2* cells (as a positive control) (Fig. 5C, *upper panels*). Bcl-2 did not co-precipitate with Ras in the mutant cells during the Fas-mediated apoptotic process, indicating that the interaction requires the intact, apoptotic signaling. The existence of Ras in the co-precipitates was confirmed by re-probing the same blots with the anti-Ras Ab (Fig. 5C, *lower panels*).

**Fig. 6.**
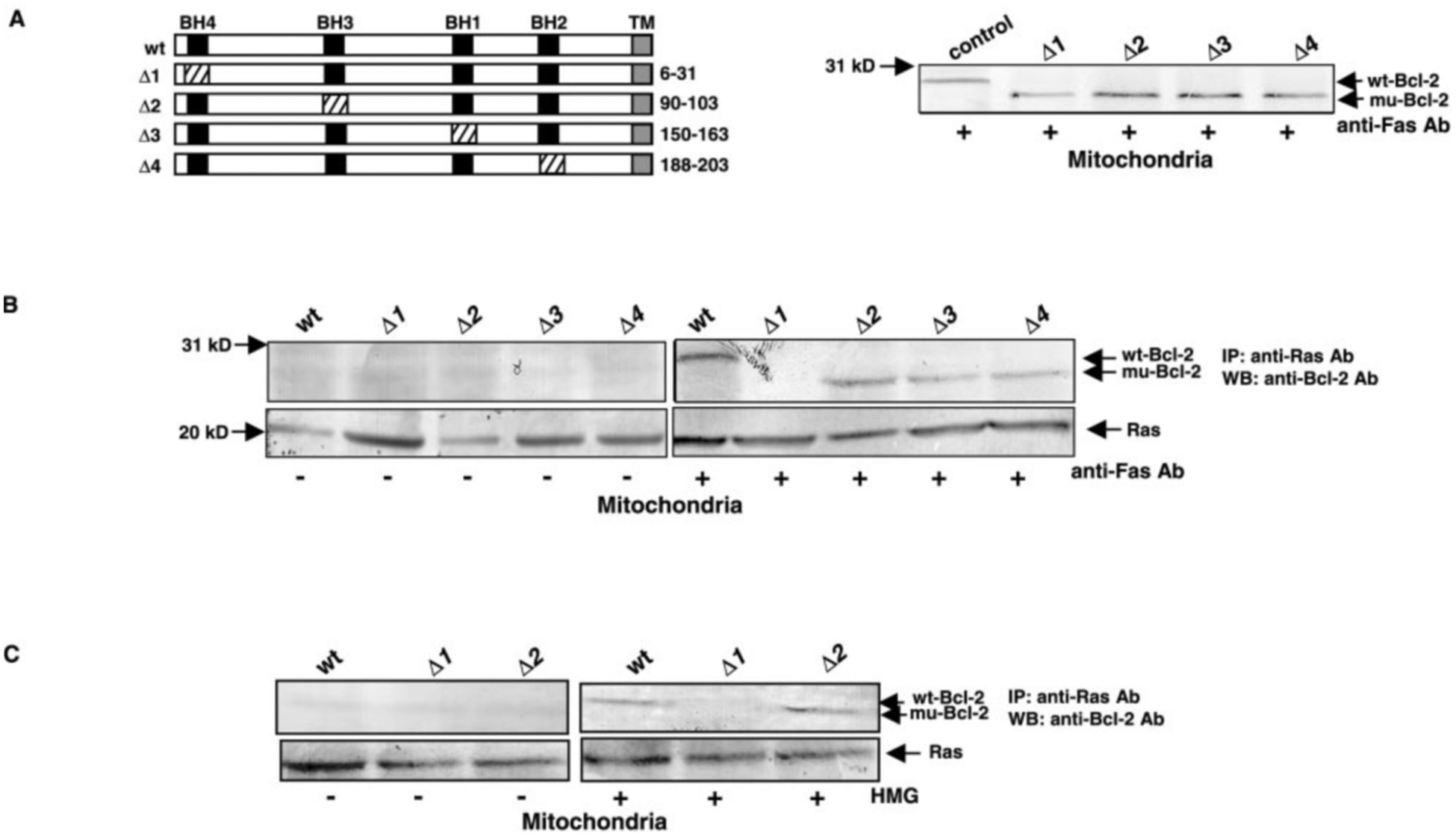
Domain analysis of Bcl-2. *A*, structures of wt- and mutants of Bcl-2 (*left panel*). For Bcl-2, the *black rectangles* represent BH domains, and the *gray region* represents the transmembrane motif (*TM*). The *cross-hatching* rectangle denotes the deletion sequence. After introducing *wt-bcl-2* or Δ-*bcl-2s* into PH1 cells, the expression of the proteins in mitochondria after Fas-ligation was examined by immunoblotting with anti-Bcl-2 Ab (*right panel*). The control represents PH1/*bcl-2* cells. *B*, mitochondrial fractions from PH1 cells expressing *wt-bcl-2* or mutant Δ-*bcl-2s*, with or without Fas-ligation, were prepared. Subsequently, samples containing equal amount of total proteins were immunoprecipitated with anti-Ras Ab, and immunoblotted with anti-Bcl-2 Ab (*upper panels*). The same blots were also re-probed with the same Ab as that used in the immunoprecipitation (*lower panels*). *C*, co-immunoprecipitation of mitochondrial Bcl-2 and Ras in PKC/Ras-mediated apoptosis. PH1 cells expressing *wt-*, Δ*1-*, and Δ*2-bcl-2* mutants were untreated or treated with HMG (150 nM) for 24 h, and the mitochondrial fractions were isolated. The samples containing equal amount of total proteins were immunoprecipitated with anti-Ras Ab and then immunoblotted with anti-Bcl-2 Ab (*upper panels*). The same blots were re-probed with the same Ab that used in the immunoprecipitation (*lower panels*).

Next, we determined the region of Bcl-2 that is responsible for the interaction. *bcl-2* mutant genes (encoding Bcl-2 proteins that contain deletions at BH4 (Δ1), BH3 (Δ2), BH1 (Δ3), and BH2 (Δ4) regions) were introduced into PH1 cells, and then the expression of the mutant proteins was examined by immunoblotting (Fig. 6A). Under normal growth conditions, Bcl-2 mutant proteins were not expressed in mitochondria (data not shown). After Fas-ligation, all Bcl-2 mutant proteins smaller than wt-Bcl-2 were present in mitochondria. The ability of Bcl-2 mutants to bind to Ras after Fas-engagement was examined by co-immunoprecipitation and immunoblotting (Fig. 6B, *upper panels*). Under normal growth conditions, there was no interaction of Bcl-2 (wild-type or mutants) with Ras in mitochondria. After Fas-ligation, wt-, Δ2-, Δ3-, and Δ4-Bcl-2 proteins co-immunoprecipitated with Ras in this subcellular compartment. However, the Δ1-Bcl-2 protein did not interact with Ras under the same conditions. A similar result was obtained from the reciprocal experiment (data not shown). The existence of Ras protein in the immunoprecipitates was determined by re-probing the same blots with the anti-Ras Ab (Fig. 6B, *lower panels*). To confirm the hypothesis that the BH4 domain of Bcl-2 is crucial for its interaction with Ras, co-immunoprecipitation and immunoblotting was conducted under PKC/Ras-mediated apoptotic conditions instead. The association of Ras with wt- or mutant-Bcl-2s in mitochondria was not seen in the untreated cells (Fig. 6C, *upper panels*). However, after HMG treatment, wt-Bcl-2 and smaller Δ2 were visualized in the Ras co-immunoprecipitate. Once again, the Δ−1 mutant protein did not co-immunoprecipitate with Ras in PKC/Ras-mediated apoptosis. The presence of Ras protein in the immunoprecipitates was also examined by re-probing the same blots with the anti-Ras Ab (Fig. 6C, *lower panels*). These data confirm that the BH4 domain of Bcl-2 is the binding region for its interaction with Ras. However, Bcl-2 that lacks the BH4 domain is still capable of redistributing to mitochondria upon induction of the death pathway.

**Fig. 7.**
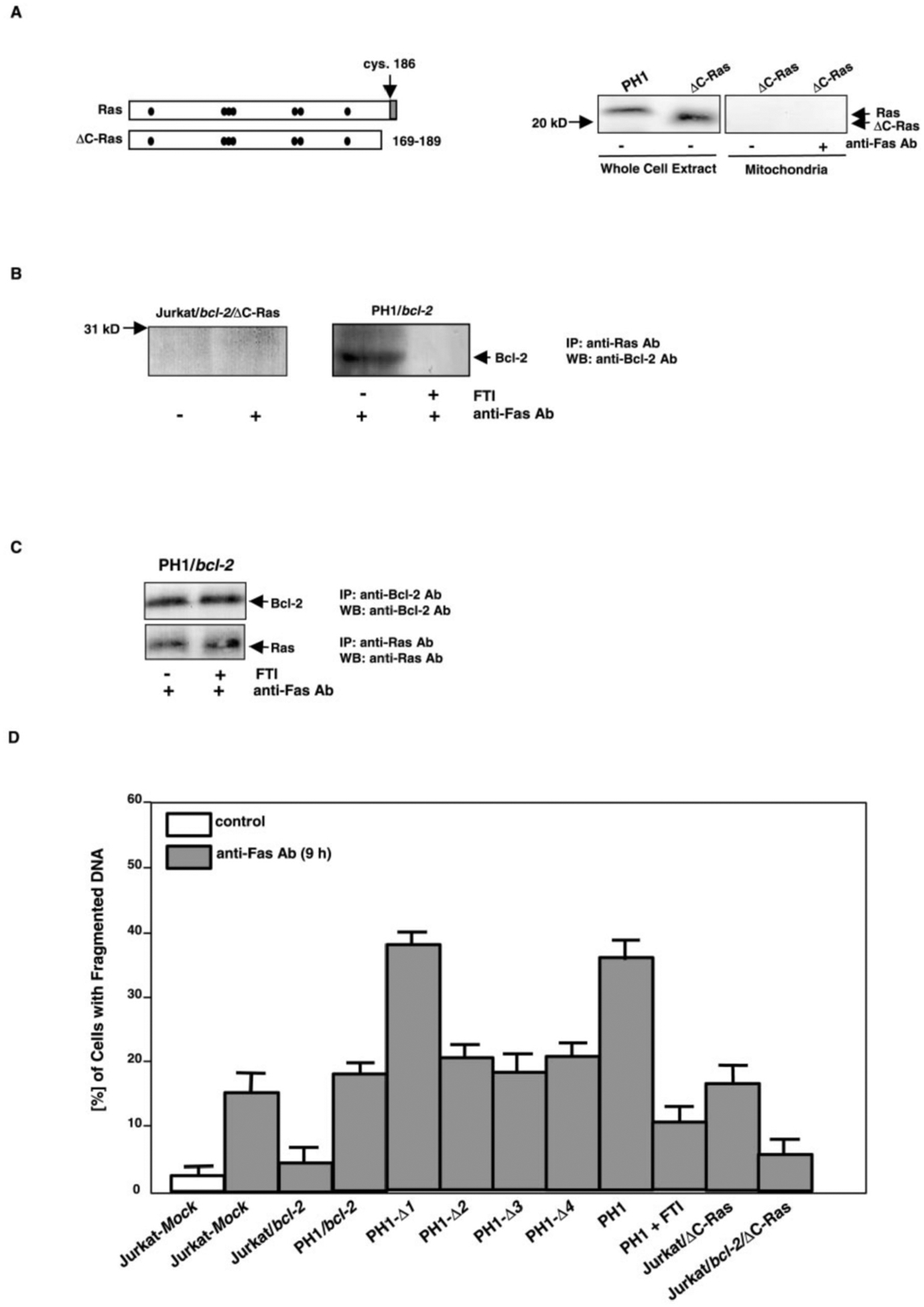
The effect of truncated Ras and of FTI on Fas-induced apoptosis. *A*, structures of Ras and C-terminal truncated Ras (*left panel*). The *black dots* represent GTP/GDP binding sites, and the *gray area* is the C*AAX* motif. PH1 and Jurkat/*bcl-2* cells that express ΔC-*ras* were treated with anti-Fas Ab for 60 min, and whole cell extracts or mitochondrial fractions were prepared (*right panels*). Samples containing equal amount of total proteins were immunoblotted with anti-Ras Ab. *B*, whole cell extracts from untreated or treated (Fas-ligation) Jurkat/*bcl-2/*ΔC-*ras* cells, or from PH1/*bcl-2* cells treated with 100 nM of FTI 12 h prior to Fas-ligation, were prepared for the co-immunoprecipitation and immunoblotting of Bcl-2 and Ras (*upper panels*). The same blots were re-probed with the same Ab as that used in the immunoprecipitation (*lower panels*). *C*, effect of FTI on co-immunoprecipitation behavior of Bcl-2 or Ras was examined using the same Ab. *D,* susceptibilities of cells that contain mutant Bcl-2s or truncated Ras- to Fas-mediated apoptosis. The mutant cells were treated with anti-Fas Ab for 9 h, and then stained with propidium iodide for DNA fragmentation assay. The *error bars* represent S.D. of five independent experiments. *Jurkat-Mock*, Jurkat cells expressing empty *MSCV* retroviral vector.

It is known that the anchor CAAX domain of Ras regulates its signaling in response to mitogenic stimulation (24–26). A C-terminal deleted Ha-*ras* (ΔC-*ras*) was introduced into Jurkat/*bcl-2* cells. The truncated Ras was detected in whole cell extract from Jurkat/*bcl-2/*ΔC-*ras* cells, with faster mobility on the gel (Fig. 7A). The expression of the mutant Ras in mitochondria was also examined, and the mutant protein could not be seen in this subcellular membrane fraction, with or without Fas-ligation. To test whether the deletion of the C terminus of Ras affects its interaction with Bcl-2 during apoptosis, whole cell extracts from untreated or Fas Ab-treated Jurkat/*bcl-2/*ΔC-*ras* cells were prepared for co-immunoprecipitation and immunoblotting. The C-terminal-deleted Ras did not bind to Bcl-2 after Fas-ligation (Fig. 7B, *left panels*). We then tested if the inhibition of the prenylation ablates the interaction of Ras with Bcl-2, using farnesyl transferase inhibitor (FTI). PH1/*bcl-2* cells were treated with FTI (100 nM) for 12 h prior to Fas-ligation, in order to block continuous farnesylation of activated Ras. Subsequently, the co-immunoprecipitation and immunoblotting was conducted in the whole cell extracts. The association of Ras with Bcl-2 in the cells was blocked by FTI (Fig. 7B, *right panels*). The existence of the mutant Ras or Ras was confirmed by re-probing the same blots with the anti-Ras Ab (data not shown). To exclude the possibility that FTI may cause a conformational change in Bcl-2 or in Ras and generate a form that can not be recognized by the corresponding Abs, co-immunoprecipitation and blotting with the same Ab was performed in PH1/*bcl-2* cells, treated or untreated with FTI prior to Fas-ligation (Fig. 7C). FTI did not alter the ability of the proteins to be immunoprecipitated. Overall, the data suggest that the prenylation of Ras is required for its interaction with Bcl-2 during the apoptotic process.

The ability of the *bcl-2* mutants to suppress Fas-induced apoptosis was examined with a DNA fragmentation assay (Fig. 7D). After 9 h of Fas-engagement, the Δ−1 mutant of Bcl-2 did not protect PH1/Δ*1* cells from apoptosis. The percentage of DNA fragmentation in the Δ*1* mutant cells was comparable to that in PH1 cells, which express Ha-*ras* alone. In contrast, the BH1 (Δ3), BH3 (Δ4), and BH2 (Δ5) deletion of Bcl-2 provided similar resistance to apoptosis as that provided by wt-Bcl-2, which is in good agreement with the observation that the pro-apoptotic factor Bax is not directly involved in Fas-induced apoptosis (39, 40). The protection provided by Bcl-2 mutants was also examined in Jurkat cells, and similar results were obtained (data not shown). We also tested whether Ras without prenylation could still transmit apoptotic signals. After addition of FTI to suppress continuous prenylation, the percentage of PH1 cells with fragmented DNA, in response to Fas-ligation, was dramatically reduced (about 3-fold). The introduction of ΔC-*ras* did not increase the susceptibility of Jurkat or Jurkat/*bcl-2* cells to Fas-induced apoptosis. We conclude that Bcl-2 may block Ras apoptotic signaling pathway in Fas-activated cells via its BH4 domain. Similar to its role in mitogenic stimulation, Ras must be prenylated to transmit its apoptotic signal.

## DISCUSSION

Jurkat cells undergo apoptosis in response to Fas-ligation and overexpressed Bcl-2 partially protects the cells from this apoptotic process (20, 22, 23, 29, 33). Ras activity is involved in the regulation of Fas-induced apoptosis (20, 29). We previously demonstrated that overexpressed Bcl-2 in Jurkat cells blocks PKC/Ras-mediated apoptosis, possibly through its association with Ras (17, 18). Experiments reported provide further insight into the mechanism by which Bcl-2 interferes with apoptotic signaling elicited by activated *ras*. The results showed that newly synthesized Bcl-2, upon Fas-ligation or PKC suppression, preferentially redistributes in the mitochondrial compartment, interacts there with Ras, and blocks Ras-mediated apoptotic signaling. In contrast, activated Ras potentiates the apoptotic process. When both Bcl-2 and Ras are overexpressed simultaneously, apoptosis once again becomes a balanced process. *bcl-2* mutants that encode the protein products lacking different BH domains demonstrate that Bcl-2 binds to activated, mitochondrial Ras through its BH4 domain, and this region is also required for its suppression of Ras-mediated apoptotic signaling. However, the deletions of the homo- or heterodimerization domains of Bcl-2, including BH4, have no effect on its redistribution mediated by either Fas-engagement or PKC inhibition. Experiments with C-terminal-truncated Ras or FTI show that deletion of the prenylation motif or inhibition of prenylation abrogate not only the apoptotic activity of Ras, but also its association with Bcl-2.

The reciprocal roles of Fas and Bcl-2 family proteins have been studied in cell lines, as well as in transgenic and mutant mice (2, 9, 22, 41). Studies suggest that Ras activity is elicited via ceramide signaling in the multiple apoptotic pathways mediated by Fas, and that overexpressed *ras* accelerates the Fas-induced death process (17, 20, 29). Conversely, as a negative regulator of apoptosis, Bcl-2 blocks cell death induced by a wide variety of effectors, and partially suppresses Fas-induced cell death (2, 22, 23, 41). In order to protect cells from apoptosis, Bcl-2 interacts with various factors and functions in several subcellular locations, particularly in mitochondria (42, 43). It is possible that, through binding to Ras, Bcl-2 neutralizes the apoptotic signaling mediated by Ras, but not other pathways, during the Fas-mediated apoptotic process. Thus, although Ras activity is involved in only one of the multiple Fas-induced death pathways, the interaction of these two proteins may affect the threshold of the sensitivity of the cells to Fas stimulation.

It has been suggested that apoptotic signaling suppresses pro-survival mechanism(s) to ensure the execution of the cell death program (44, 45). For example, cross-linking of Fas antigen inhibits some of the TcR/CD3-mediated signals, as well as the activation of PKC*θ* and PKC*ϵ* (44, 45). In LKR cells, although endogenous Bcl-2 redistributes to mitochondria, there is little protection against Ras-mediated apoptosis. Furthermore, in PH1 cells, endogenous Bcl-2 does not co-immunoprecipitate with Ras during Fas-induced apoptosis, and further protects these cells. Because Bcl-2 requires modification (such as phosphorylation) to function (18, 46), it is possible that Ras-mediated apoptotic signaling activates a phosphatase that regulates Bcl-2 phosphorylation status, and further inhibits the anti-apoptotic activity of Bcl-2.

The C terminus of Bcl-2 contains a stretch of hydrophobic amino acids that anchors the protein in the lipid bilayer of the membrane compartments (4–6). Bcl-2 that lacks the C-terminal domain loses the ability to associate with cellular membranes and to suppress cell death (47, 48). Some Bcl-2 family members, such as Bcl-X_L_ or Bax, undergo subcellular redistribution in response to various apoptotic stimuli (38). Here, we demonstrate that overexpressed Bcl-2 is located in subcellular membrane compartments, mainly in the ER. Upon Fas-ligation or PKC suppression, Bcl-2 or its BH domain deletion mutants preferentially express in mitochondria. The results suggest that subcellular redistribution of Bcl-2 is an early event during the apoptotic process. This process only requires the C-terminal anchor region of Bcl-2, not the protein/protein interaction domains. Given the fact that the mitochondrial membrane has been identified as one of the Bcl-2 targets and that increases in mitochondrial free radicals have been observed in apoptosis mediated by oncogenic Ras (10, 42, 43, 49), it is reasonable to propose that apoptotic signals redirect Bcl-2 to damaged sites. In this case, Bcl-2 mediated by Fas signaling or PKC inhibition, is preferentially expressed mitochondria, and subsequently reduces or blocks an increase in mitochondrial permeability, thereby preventing the Ras-triggered release of cytochrome *c* or free radicals.

It is well established that subcellular targeting of Ras protein requires multiple steps of post-translational modification at its C-terminal C*AAX* motif in response to growth or differentiation stimulation, such as prenylation (24–26). Upon mitogenic or growth stimulation, prenylated Ras translocates from cytosol to subcellular membrane compartments, mainly to plasma membrane. However, it is not clear whether Ras requires this modification for its apoptotic activity. We show here that inhibition of the prenylation of activated Ras interferes with its binding to Bcl-2, as well as its apoptotic activity. We also demonstrate that overexpressed, activated Ras proteins localize normally in various intracellular membrane compartments and do not undergo redistribution or translocation upon apoptotic stimulation. Therefore, we speculate that activated Ras in different locations may have different roles in biological processes, depending upon the nature of the stimulation or the context of the signaling transmitters. Instead of its traditionally known effectors (such as Raf-1) that transmit growth or differentiation signals from cytoplasmic membrane to nucleus, after apoptotic stimulation, mitochondrial Ras may interact with a different set of signal transducers to execute the death program.

Many studies have suggested the involvement of multiple pathways in Fas-mediated apoptosis (17, 20, 29). In response to Fas-ligation, FADD binds and recruits caspase 8 to form the receptor complex, which ultimately results in the activation of Fas apoptotic signaling (50–53). Experiments from Fas or FADD mutant cells or from knockout mice show the importance of these molecules in the induction of cell death (54–56). Studies have also demonstrated that upon Fas-engagement, molecules such as DAXX bind to Fas and mediate FADD-independent apoptotic signaling (57). The results of experiments with Fas- and FADD-deficient mutant cell lines indicate that intact Fas and FADD are required for the interaction between Ras and Bcl-2, and that Ras is a downstream effector of Fas and FADD during Fas-induced apoptosis. However, these results do not rule out the existence of parallel signaling pathways that may originate from Fas-ligation, but that are not sufficient to elicit the interaction or are independent of Ras activity.

During activation-induced T lymphocyte death, a correlation has been observed between the expression of *bcl-2* and *fas* genes. Upon priming, the expression of *bcl-2* in a T cell progressively falls and the expression of Fas reciprocally increases (58, 59). Our study demonstrates that oncogenic Ras accelerates Fas-mediated apoptosis in Jurkat cells, whereas overexpressed Bcl-2 confers resistance to the death process. Neither the overexpression of Bcl-2 nor oncogenic Ras changes cell surface levels of Fas antigen expression; therefore, facilitation of Fas-induced apoptosis by activated Ras (and resistance to the death process by forced Bcl-2 expression) is likely to operate at an essential step in the Fas-mediated signal transduction pathway that is distal to Fas ligand expression.

Our study provides evidence for a model of the dynamic interrelationship between Bcl-2 and Ras molecules: in response to mitogenic stimuli, activated Ras mainly located at the plasma membrane transmits signals to downstream effectors and mobilizes the growth program. Upon death stimulation, however, apoptotic factors elicit mitochondrial Ras activity, a process which Bcl-2 blocks. We define specific domains of Bcl-2 and Ras that mediate this interaction. In particular, the BH4 domain of Bcl-2 controls both its association with Ras and protection against Ras-induced apoptotic signals. We speculate therefore that naturally occurring BH4 domain mutants of Bcl-2 may be linked to certain defects in T lymphocyte cell death. An increased understanding of the pathways regulated individually or collectively by these mediators may guide approaches to Fas- or Ras-based tumor immunotherapy.

## abbreviations:

ER: endoplasmic reticulum
PKC: protein kinase C
Ab: antibody
wt: wild type
FTI: farnesyl transferase inhibitor
C*AAX*: motif where *A* is an aliphatic residue and *X* is any amino acid
HMG: 1-*O*-hexadecyl-2-*O*-methyl-*rac*-glycerol
DISC: death-inducing signaling complex
BH: Bcl-2 homology
FADD: Fas-associated death domain
